# The highly continuous reference genome of a leaf-chimeric red pineapple (*Ananas comosus* var. *bracteatus* f. *tricolor*) provides insights into elaboration of leaf color

**DOI:** 10.1093/g3journal/jkab452

**Published:** 2022-01-09

**Authors:** Lijun Feng, Juntao Wang, Meiqin Mao, Wei Yang, Mark Owusu Adje, Yanbin Xue, Xuzixin Zhou, Huiling Zhang, Jiaheng Luo, Ruimin Tang, Lin Tan, Dongpu Lin, Xiaopeng Zhang, Yaoqiang Zang, Yehua He, Changming Chen, Aiping Luan, Wenqiu Lin, Wentian Xu, Xi Li, Lingxia Sun, Fuxing Jiang, Jun Ma

**Affiliations:** 1 College of Landscape Architecture, Sichuan Agricultural University, Chengdu, Sichuan 611130, China; 2 College of Horticulture, South China Agricultural University, Guangzhou, Guangdong 510642, China; 3 Tropical Crops Genetic Resources Institute of Chinese Academy of Tropical Agricultural Science, Haikou, Hainan 571101, China; 4 South Subtropical Crop Research Institute, China Academy of Tropical Agricultural Sciences, Zhanjiang, Guangdong 524000, China

**Keywords:** *Ananas comosus* var. *bracteatus* f. tricolor, assembly, genetic variation, anthocyanin biosynthesis genes, photosynthesis genes

## Abstract

*Ananas comosus* var. *bracteatus* f*. tricolor* (GL1) is a red pineapple accession whose mostly green leaves with chimeric white leaf margins turn red in spring and autumn and during flowering. It is an important ornamental plant and ideal plant research model for anthocyanin metabolism, chimeric leaf development, and photosynthesis. Here, we generated a highly contiguous chromosome-scale genome assembly for GL1 and compared it with other 3 published pineapple assemblies (var. *comosus* accessions MD2 and F153, and var. *bracteatus* accession CB5). The GL1 assembly has a total size of ∼461 Mb, with a contig N50 of ∼2.97 Mb and Benchmarking Universal Single-Copy Ortholog score of 97.3%. More than 99% of the contigs are anchored to 25 pseudochromosomes. Compared with the other 3 published pineapple assemblies, the GL1 assembly was confirmed to be more continuous. Our evolutionary analysis showed that the Bromeliaceae and Poaceae diverged from their nearest common ancestor ∼82.36 million years ago (MYA). Population structure analysis showed that while GL1 has not undergone admixture, *bracteatus* accession CB5 has resulted from admixture of 3 species of *Ananas*. Through classification of orthogroups, analysis of genes under positive selection, and analysis of presence/absence variants, we identified a series of genes related to anthocyanin metabolism and development of chimeric leaves. The structure and evolution of these genes were compared among the published pineapple assemblies with reveal candidate genes for these traits. The GL1 genome assembly and its comparisons with other 3 pineapple genome assemblies provide a valuable resource for the genetic improvement of pineapple and serve as a model for understanding the genomic basis of important traits in different pineapple varieties and other pan-cereal crops.

## Introduction

*Ananas comosus* var. *bracteatus* f*. tricolor* GL1 (2*n* = 50) is a red pineapple accession with green leaves with white leaf margins that is cultivated in tropical regions. Because the fruit and bracts of var. *bracteatus* accessions are deep red, whereas those of var. *comosus* are green or yellow, var. *bracteatus* accessions are grown as ornamental plants. Compared with var. *bracteatus* accession CB5, the genome of which has been sequenced and analyzed (Chen et al. 2019), GL1 bears red fruit and bracts, as well as chimeric green and white striped leaves that change to deep red during spring, autumn and flowering. These unique color characteristics of its leaves, bracts, and fruit make GL1 a very important ornamental plant. The red coloration of the leaves, bracts, and fruit is caused by the accumulation of anthocyanins (Zhou et al. 2021). Reports show that the accumulation of anthocyanins is closely related to resistance to biotic stress due to insects and diseases, and abiotic stress such as high light or low temperature (Sarma et al. 1997; Gould and Quinn 1999; Ahmed et al. 2015). The decreased chlorophyll content and incomplete chloroplast development of the chimeric leaves of GL1 result in changes in photosynthesis (Li et al. 2017; Xue et al. 2019; Mao et al. 2020; Zhou et al. 2021). Similar to most pineapple [*A.* *comosus* (L.) *Merr.*] varieties, GL1 is a crassulacean acid metabolism crop with higher drought resistance (Yang et al. 2015). Thus, GL1 is an important ornamental plant and ideal research model for the study of anthocyanin metabolism, chimeric leaf development, photosynthesis, and stress resistance.

Pineapple species, including GL1, have a close phylogenetic relationship with pan-cereal crops such as rice (*Oryza sativa* L.) and sorghum [*Sorghum bicolor* (L.) Moench], thereby pineapple may serve as a good outgroup for genomic analysis of other pan-cereal crops (Ming et al. 2015). In recent years, the frequency of natural disasters such as drought, low temperature, and insect pests has increased significantly cause significant damage to the production of important crops. Thus, studying the genomic basis of traits related to photosynthesis and stress resistance in various pineapple varieties is important. Further, it may be helpful to modulate the anthocyanin content and way of photosynthesis of pineapple and other pan-cereal crops to increase resistance to stress.

Due to the high-heterozygosity and high-repetitive DNA content of the genome of pineapple species, high-quality reference genomes for pineapple varieties are essential for further study of the unique characteristics of *Ananas* species. Pineapple genome resources are available for 1 var. *bracteatus* accession (CB5) and 2 var. *comosus* accessions (F153 and MD2) (Ming et al. 2015; Redwan et al. 2016, p. 2; Chen et al. 2019). Compared with the published pineapple varieties, GL1 exhibits leaf and fruit coloration. The lack of a reference genome for this accession hindered studies of the mechanisms of the development of the unique leaf and fruit color characters and high stress resistance of GL1. In this study, a combination of high-depth Pacific Biosciences (PacBio) sequencing (∼285×), Illumina sequencing (∼156×), and Hi-C sequencing technology (∼211×) were performed to generate a high-quality chromosome-scale de novo assembly of the GL1 genome. To elucidate genetic characteristics related to important traits of various pineapple varieties at the genome level, we performed phylogenetic analysis, population genetic analysis, and genome variation analysis of GL1 and other pineapple varieties and identified a series of genes related to anthocyanin metabolism, photosynthesis, hormone response, and defense response. These results are valuable resource for analysis of genetic diversity and the improvement of pineapple in breeding programs for these related species and other pan-cereal crops.

## Materials and methods

### Sample collection

Genome sequencing and assembly was performed on *A.* *comosus* var. *bracteatus* f. *tricolor* (accession number GL1), which has green and white chimeric leaves, with red fruit and bracts. These plants were cultivated in the greenhouse at Sichuan Agricultural University in Chengdu, Sichuan.

### Genome sequencing and assembly

Before sequencing for genome assembly, a survey analysis was first carried out to estimate the genome profile of GL1. High-quality genomic DNA was extracted from leaves using a modified CTAB method (Porebski et al. 1997). The quality and the quantity of the extracted DNA were examined using a NanoDrop 2000 spectrophotometer (NanoDrop Technologies, Wilmington, DE, USA), a Qubit dsDNA HS Assay Kit with a Qubit 3.0 Fluorometer (Life Technologies, Carlsbad, CA, USA), and electrophoresis on a 0.8% agarose gel. Sequencing for this genome survey was conducted on an Illumina HiSeq 2000 platform (Illumina, USA) with an insert length of 350 bp. After filtering out low-quality reads using the HTQC package v0.90.8 (Yang et al. 2013), the genome size, heterozygosity, and repeat content were estimated based on the k-mer method using Jellyfish v2.2.3 (Marçais and Kingsford 2011) with a k-mer size of 17. Genome sequencing for genome assembly was then performed. Genomic DNA (10 μg) extracted from GL1 chimeric leaf sample was used to prepare a 30-kb template library using the BluePippin Size Selection System (Sage Science, USA). The library was sequenced to generate long genomic reads on the PacBio SEQUEL II platform (PacBio, USA). After removing adaptor sequences, more than 138 Gb of subreads were obtained with ∼286× sequence coverage. Illumina short reads sequencing and quality control were taken by the same method as survey sequencing. Then, 8 g of young leaf tissue collected from GL1 was used for Hi-C library construction and sequenced by Illumina HiSeq 2000 platform (Illumina). Sequencing details are shown in Supplementary Table 1.

The MECAT2 package (Xiao et al. 2017) was used for the initial de novo genome assembly with a length cutoff of 20 kb for long reads. Then, 2 rounds of polishing using NGS short reads with Pilon v1.23 (Walker et al. 2014) were applied. Contigs with a series repeat ratio >60% identified by TRF v4.09 (Benson 1999) were removed. Then, Purge_haplotigs v1.1.1 (Roach et al. 2018) were used to remove the redundant heterozygous contigs after mapping the long reads to the draft assembly using Minimap2 v2.10 (Li 2018). Juicer (Durand et al. 2016) was used to analyze the Hi-C data and 3D-DNA (Durand et al. 2016) to break down spurious contigs. ALLHIC (Zhang et al. 2018) was used to anchor the contigs into a superscaffold including 25 pseudochromosomes. BWA v 0.7.15 (Li 2013) was used to map the paired-end reads to the assembly and Benchmarking Universal Single-Copy Ortholog (BUSCO) v3.0 (Simão et al. 2015) was run with embryophyta_odb10 to evaluate the integrity and conservation of the assembly.

### Repeat sequence annotation

Referring to the method used for annotating the CB5 genome (Chen et al. 2019), RepeatModeler v2.0.1 (http://www.repeatmasker.org) was used to generate a de novo transposable element (TE) library. Unknown TEs were further classified using TEclass v2.1.3 (Abrusan et al. 2009). Consensus TE sequences generated above were imported to RepeatMasker v4.09 (http://www.repeatmasker.org) to identify and cluster repetitive elements. The TRF package was used with the modified parameters of “1 1 2 80 5 200 2000 –d -h” in order to identify tandem repeats. LTR_finder v1.0.7 (Xu and Wang 2007) and LTR_harvest in Genometools v1.2.1 (Gremme et al. 2013) were used to identify long-terminal repeat (LTR) sequences in the genome. Then the results from the 2 software were imported to LTR_retriever v2.9.0 (Ou and Jiang 2018) to identify high-quality, full-length LTR sequences, and calculate their insertion times.

### Annotation of protein-coding genes and noncoding RNAs

In order to obtain more complete gene annotations to allow us to predict the genes encoding GL1 proteins, we combined full-length transcript data, RNA-seq data, homologous proteins sequence data, and ab initio gene prediction data. Full-length transcript sequences consisted of nonredundant isoforms from our previous IsoSeq experiment (Ma et al. 2019). Information regarding these isoforms is available in Supplementary File 1 and detailed information about the IsoSeq experiment can be found in NCBI bioproject PRJNA494788. The RNA-seq data were also obtained from our previous study (Xue et al. 2019) (data are available at ProteomeXchange, project accession number: PXD010375). RNA-seq data were used to generate a de novo transcriptome assembly with Trinity v2.12.0 (Haas et al. 2013). The protein evidence used for gene annotation came from *Arabidopsis thaliana* (TAIR10) and *O.* *sativa* L. (IRGSP-1.0) downloaded from Ensemble (Yates et al. 2020), and from var. *comosus* accession F153 (http://pineapple.angiosperms.org) and var. *bracteatus* accession number CB5 (https://www.life.illinois.edu/ming/LabWebPage/Downloads.html) (Chen et al. 2019). All transcript sequences, protein sequences, and the soft-masked GL1 genome sequences were imported into the MAKER pipeline v2.31.9 (Cantarel et al. 2008, p. 2) for 5 rounds of gene prediction.

For the functional annotation of genes putatively encoding GL1 proteins, BLASTp v2.9.0 (Altschul et al. 1990) was used to compare each candidate protein sequence with SwissProt protein sequences (Bairoch and Apweiler 2000) and the NCBI nonredundant protein database (E-value ≤ 1e−5). InterProScan (Mulder and Apweiler 2008; Jones et al. 2014) was used to annotate conserved domains and gene ontology (GO) terms in candidate proteins. KEGG pathway terms were annotated using the KEGG Automatic Annotation Server website (https://www.genome.jp/kegg/kaas/). Transfer RNAs (tRNAs) were predicted using tRNAscan-SE v2.0.7 (Lowe and Eddy 1997). Ribosomal RNA (rRNA) genes were annotated using Barrnap software (https://github.com/tseemann/barrnap). MicroRNAs and small nuclear RNAs were identified by searching against the Rfam database (Griffiths-Jones 2003) with default parameters using INFERNAL software v1.1.4 (Nawrocki and Eddy 2013).

### Comparative genome analysis

OrthoFinder v2.5.1 (Emms and Kelly 2015) was used to classify proteins from 4 pineapple varieties (GL1, CB5, F153, and MD2) and other 9 representative plants (Supplementary Table 10) into orthogroups. A total of 499 single-copy gene families shared among 13 species were used to perform phylogenetic analysis (Supplementary Table 11 and File 2). Multiple sequence alignments were performed using MUSCLE v3.8.31 (Edgar 2004), and RAXML v8.2.12 (Stamatakis 2014) was used to construct a phylogenetic tree based on the Maximum Likelihood method with 2,000 bootstraps. The calibrated time for each node was set using the timetree website (timetree.org) (Kumar et al. 2017), and Mcmctree in the PAML software package v4.9 (Inoue et al. 2011) was used to estimate the divergence times.

The ratios of nonsynonymous to synonymous substitutions (Ka/Ks) were calculated using the Codeml program with the free-ratio model implemented in PAML. Analysis of positive selection was performed using the Codeml program with the optimized branch site model as implemented in the PAML package.

### Population genetics analysis

Three resequenced GL1 samples and 28 previously resequenced pineapple samples in CB5 genome (Chen et al. 2019) were used for population genetic analysis. Detailed information for these samples is shown in Supplementary Table 16. Qualified NGS reads for each sample were aligned to the GL1 genome using the BWA v 0.7.15 (Li 2013). The resulting bam file was then used following GATK4 best practices for detecting mutations (DePristo et al. 2011). First, MarkDuplicates was used to mark duplicate fragments, and then HaplotypeCaller was used to perform variant calling for each sample. Individual genome Variant Call Format (gVCF) files were merged using CombineGVCFs and genotyped using GenotypeGVCFs. Then, SelectVariants was used to extract single nucleotide polymorphism (SNP) and insertion/deletion polymorphism (INDEL) information, respectively. VariantFiltration was used to filter out low-quality SNPs and INDELs. The parameter used for filtering SNPs was “QD < 2.0 ‖ FS > 60.0 ‖ SOR >3.0 ‖MQ <40.0 ‖ MQRankSum <-12.5,” and for INDELs was “QD < 2.0 ‖ FS > 200.0 ‖ SOR >10.0 ‖MQ <40.0 ‖ MQRankSum <-12.5.” VCFtools v 0.1.16 (Danecek et al. 2011) with the parameters “–recode –recode-INFO-all –stdout –max-missing 0.85 –maf 0.05 –minDP 4” was used to further filter out low-quality SNPs.

Based on the SNP information (Supplementary File 3), population structure was then analyzed using Admixture v1.3.0 (Alexander et al. 2009). Samples with no admixture were used for further analysis. VCFtools and Plink software v 1.90b5 (Purcell et al. 2007) were used for principal component analysis (PCA). The SNP information was also used to construct a phylogenetic tree with TreeBest software (http://treesoft.sourceforge.net/index.shtml) based on the neighbor joining method with 2,000 bootstraps.

### Identification of SNPs, INDELs, and presence/absence variants among genomes of F153, CB5, and GL1

Following methods published for the *Brassica rapa* L. subsp. *chinensis* genome (Li et al. 2021) and the *Zea mays* genome (Sun et al. 2018), 3 chromosome-level pineapple genomes were used to perform genomic variant analysis. SNPs and INDELs (length < 100 bp) were identified using Mummer (Kurtz et al. 2004) by comparing the GL1 genome to those of CB5 and F153. Specifically, the GL1 genome was mapped to its corresponding CB5 sequences using nucmer with the parameters “-mumreference -g 1000 -c 90 -l 40.” The delta-filter was then used to reduce mapping noise and identify the 1-to-1 alignment blocks with parameters “-r -q.” Show-snps was then used to identify SNPs and small INDELs (<100 bp). CB5 genome-based SNPs and INDELs were detected using the parameter “-ClrTH,” and GL1 genome-based parameters were detected with the parameter “-ClqTH.” Further, all clean GL1 Illumina reads were mapped to the CB5 genome using BWA-MEM (Li 2013). The GATK pipeline and VCFtools were then used for variant calling and filtered using the same parameters shown above for filtering variants for population genetics analysis. Only variants detected by both tools were considered high-quality variants. The same method was used to identify SNPs and INDELs between GL1 and F153 genome.

We used a sliding-window approach to identify the presence/absence variations (PAVs) among the 3 pineapple genomes. To identify GL1-specific sequences, the GL1 genome was first divided into 500-bp overlapping windows with a step size of 100 bp. BWA-MEM (Li 2013) was then applied to map each window against the CB5 genome, with parameter settings of “-w 500 –M.” The sequences of windows that could not be mapped or that mapped to the CB5 genome with a primary alignment coverage of <25% were defined as GL1-specific sequences. The coding DNA sequence (CDS) of different transcripts from individual genes were merged to represent a single gene, and genes with more than 75% of their CDS regions covered by PAV sequences were defined as PAV genes. CB5 and F153 specific sequences and PAV genes were determined using the same method. To validate the PAV variants, we further aligned long reads of GL1 to the CB5 and F153 genome using NGMLR mapper (NGMLR; https://github.com/philres/ngmlr), and variants were called using Sniffles with a minimum read depth of 10 (Sedlazeck et al. 2018). GO annotations were performed for the 3 published pineapple genomes using InterProScan (Mulder and Apweiler 2008; Jones et al. 2014). GO term enrichment analysis was performed for all specific genes (unique orthogroups, positively selected genes, and PAV genes) in each pineapple genome using the R package clusterProfiler (Yu 2018). GO terms showing a raw *P*-value < 0.05 were considered significantly enriched.

## Results and discussion

### Genome assembly

**Fig. 1.**
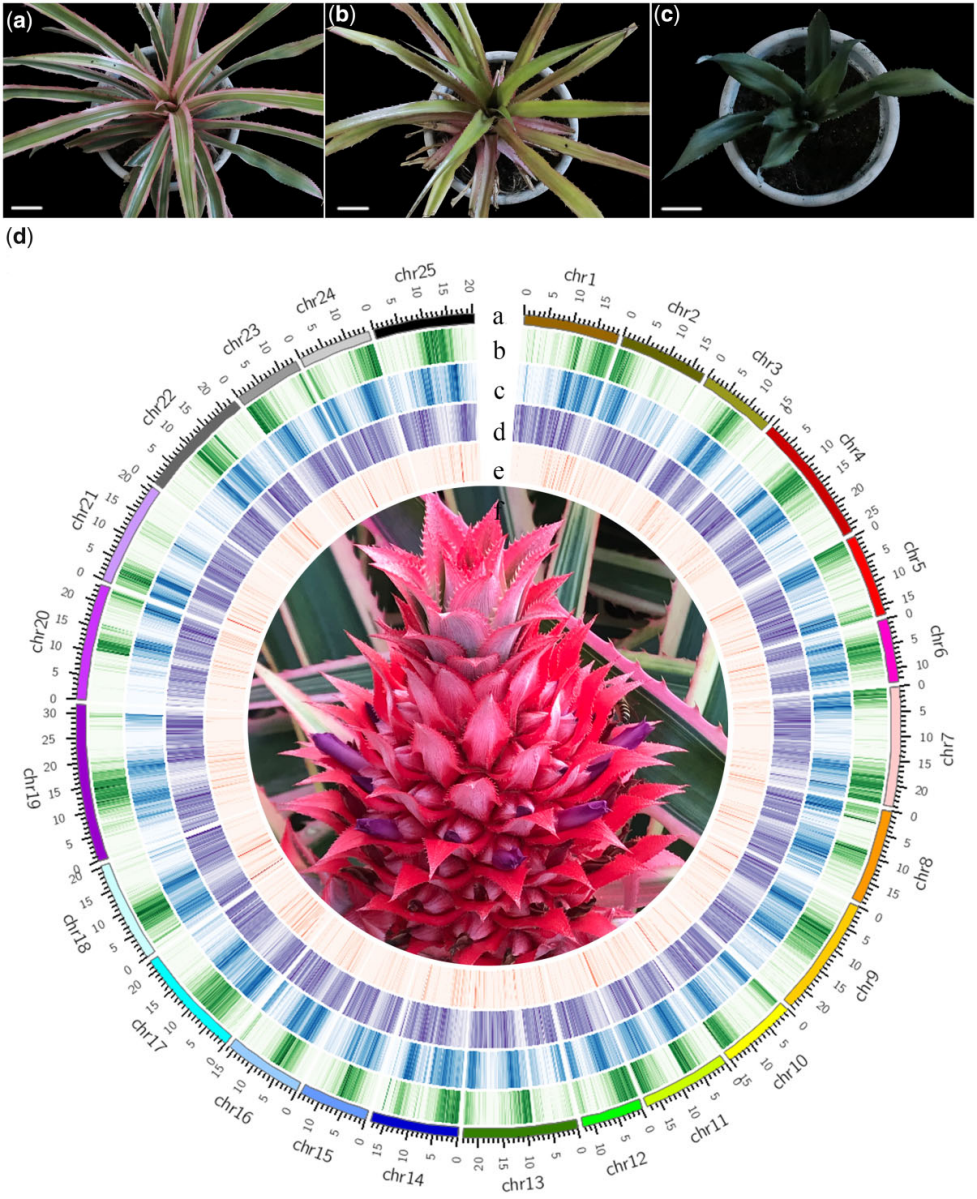
Leaf and bract characteristics and genome profile of *A. comosus* var. *bracteatus* f. *tricolor* pineapple accession GL1. a) GL1 plant with chimeric leaves; b) a green plant generated from the stem of a GL1 plant by tissue culture that lost the green and white striped chimeric character of the leaves; c) CB5 plant; d) overview of the GL1 genome: (a) the 25 pseudochromosomes of the GL1 assembly; (b) gene density; (c) DNA-type TE density; (d) long-terminal repeat (LTR) density; (e) tandem repeat density; (f) GL1 fruit [the scale bar in (a, b, and c) represents 5 cm. All of the genomic characteristics in (d) were calculated in 100-kb sliding windows].

The GL1 pineapple accession has green and white striped chimeric leaves that turn red in the spring and autumn and during flowering (Fig. 1a). When using GL1 chimeric plants as explants for tissue culture, a completely green plant could be regenerated (Fig. 1b) (Li et al. 2017*;*Xiong et al. 2018); a leaf from this completely green plant was used as a sample for resequencing in the present study. In contrast, the leaves of var. *bracteatus* accession CB5 are completely green without white stripes, which is the main difference between the leaves of GL1 and CB5 accessions (Fig. 1c) (Chen et al. 2019). Our genomic survey revealed a heterozygosity rate of 2.08%, which indicates that the GL1 genome is highly heterozygous (Supplementary Fig. 1 and Table 2). We combined high-depth PacBio long reads (∼286×), Illumina short-reads(∼157×), and Hi-C sequencing (∼212×) to perform de novo assembly of the GL1 genome (Supplementary Table 1). After de novo assembly of the PacBio reads and improvement of the preliminary assembly with Illumina short reads, we obtained a draft genome of ∼801 Mb. Due to the high heterozygosity of the GL1 genome, we identified more than 300 Mb of redundant contigs. After removing the redundant heterozygous sequences, the genome was anchored into 37 scaffolds with 25 pseudochromosomes ranging from 11.89 to 30.41 Mb in length (Supplementary Table 3). The final assembly size of the genome was ∼461 Mb, with a contig N50 of 3.12 Mb and scaffold N50 of 19.44 Mb. The 25 pseudochromosomes have a total size of 460.74 Mb and occupy more than 99% of the genome (Fig. 1d and Table 1; Supplementary Table 4). The Hi-C linkage map for the whole assembly and each pseudochromosome of GL1 are shown in Supplementary Figs. 2 and 3, respectively.

**Table 1.** Comparison of the assembly quality of *Ananas* accessions GL1, CB5, F153, and MD2.

Accession	GL1	CB5	F153	MD2
Genome size	461 Mb	498 Mb	377 Mb	500 Mb
Contig N50	2.97 Mb	0.42 Mb	0.02 Mb	0.05 Mb
Scaffold N50	19.43 Mb	19.24 Mb	11.21 Mb	0.15 Mb
BUSCO score	97.3%	92.6%	97.2%	97.6%
Gap N	52,408 bp	186,700 bp	6,791,306 bp	14,321,016 bp

**Table 2.** Statistics for assembly and annotation of the *A. comosus* var. *bracteatus* f. *tricolor* accession GL1 genome.

Parameters	Value
Assembly features	
Genome size of assembly	∼461 Mb
Number of contigs	516
Contigs N50	2.97 Mb
Longest contig	8.01 Mb
Number of scaffolds	37
Scaffolds N50	19.43 Mb
Longest scaffold	30.41 Mb
Number of complete BUSCOs	1,571
Percentage of complete BUSCOs	97.3%
GC content	39.86%
Number of gap N	52,408 bp
Genome annotation	
Total repetitive sequences	314.27 Mb
Proportion of total repetitive sequences	68.20%
Number of protein-coding genes	26,113
Average gene length	3,748.08 bp
Average number of exons per gene	5.22
Average coding region length	1,059.57 bp
Gene density per 100 kb	5.42 kb

The completeness and the accuracy of the assembly were assessed using 2 approaches. First, we used BUSCOs (Simão et al. 2015) method to annotate the core eukaryotic genes present in the GL1 assembly. We detected 1,571 complete BUSCOs, or 97.3% of the total set of 1,614 BUSCOs. Single and multicopy genes accounted for 95.5% and 1.8% of the complete BUSCOs, respectively (Supplementary Table 5). Second, the assembled genome was aligned with the Illumina short-reads. The mapping rate for these short reads was 93.69% with mapping coverages of >4×, 10×, and 20× or 99.42%, 99.09%, and 98.6%, respectively (Supplementary Table 6).

### Assembly quality comparison

To further evaluate the quality of the GL1 genome assembly, we compared it with 3 other pineapple genomes including 2 var. *comosus* accessions (MD2 and F153) and the var. *bracteatus* accession CB5.

As shown in Table 1, the scaffold N50 of the MD2 assembly is ∼0.15 Mb and the number of N gap value is as high as ∼14 Mb, which is not a chromosome-level assembly. Each of the other 3 pineapple assemblies is chromosome-level with scaffold N50 ranging from 11.21 Mb to 19.43 Mb. The assembly size of GL1 is ∼461 Mb, which is close to that of the CB5 (498 Mb) assembly but larger than the F153 assembly (377 Mb). Considering assembly continuity, the contig N50 of GL1 is 2.97 Mb, which is 7.07 times that of CB5 and 148.5 times that of F153. In addition, the N gap value of GL1 is ∼52 kb, which is significantly lower than those of CB5 (∼186 kb), F153 (∼6.79 Mb), and MD2 (∼14.3 Mb). Among the 3 chromosome-level pineapple assemblies, the GL1 assembly has 1,571 complete BUSCOs or 97.3% of the complete set of 1,614 BUSCOs, a higher percentage than identified in the CB5 (92.6%) or F153 (97.2%) assemblies. These results indicate that the GL1 assembly is the most continuous and conservative of the pineapple genome sequences.

### Genome annotation

TEs and other repeat sequences are widely dispersed in plant genomes (Maumus and Quesneville 2016). Our annotation of repetitive sequences revealed that 68.20% of the GL1 assembly is annotated as repetitive elements, including DNA transposons (47.78%), retrotransposons (13.18%), tandem repeat sequences (0.41%), and unclassified elements (6.83%). The most abundant repeat elements in the GL1 assembly are LTR retrotransposons, which account for 44.80% of the genome. Within the LTR-type repetitive elements, *Gypsy* elements account for 21.29% and *Copia* elements account for 8.23% of the genome (Table 2 and Fig. 1d; Supplementary Table 7). To examine transposon activity, a total of 5,021, 3,634, and 609 full-length LTR retrotransposons were identified in the GL1, CB5, and F153 assemblies, respectively. The lower number of full-length LTR retrotransposons in F153 may have been caused by highly similar sequences collapsing when assembled from short reads (Supplementary Table 8). In addition, our LTR insertion time analysis revealed that the expansion of LTR retrotransposons occurred mainly within the past million years in both the GL1 and CB5 genomes. The LTR burst in CB5 occurred ∼1.7–1.8 MYA and the LTR burst in GL1 took place ∼2.0–2.1 MYA (Supplementary Fig. 4).

By combining full-length transcripts, de novo assembly transcripts, homologous protein sequences, and ab initio gene prediction methods, a total of 26,113 protein-coding genes were annotated in the GL1 genome assembly. We found that in this genome the gene density per 100 kilobases (kb) is 5.42, average gene length is 3,747.08 bp, average number of exons per gene is 5.42, and the average length of coding region per gene is 1,059.57 bp. In addition, a total of 379 tRNAs, 143 microRNAs, 195 small nuclear RNAs, and 95 ribosomal RNAs were identified in the GL1 genome assembly (Table 2; Supplementary Table 9).

### Phylogenetic evolutionary analysis

**Fig. 2.**
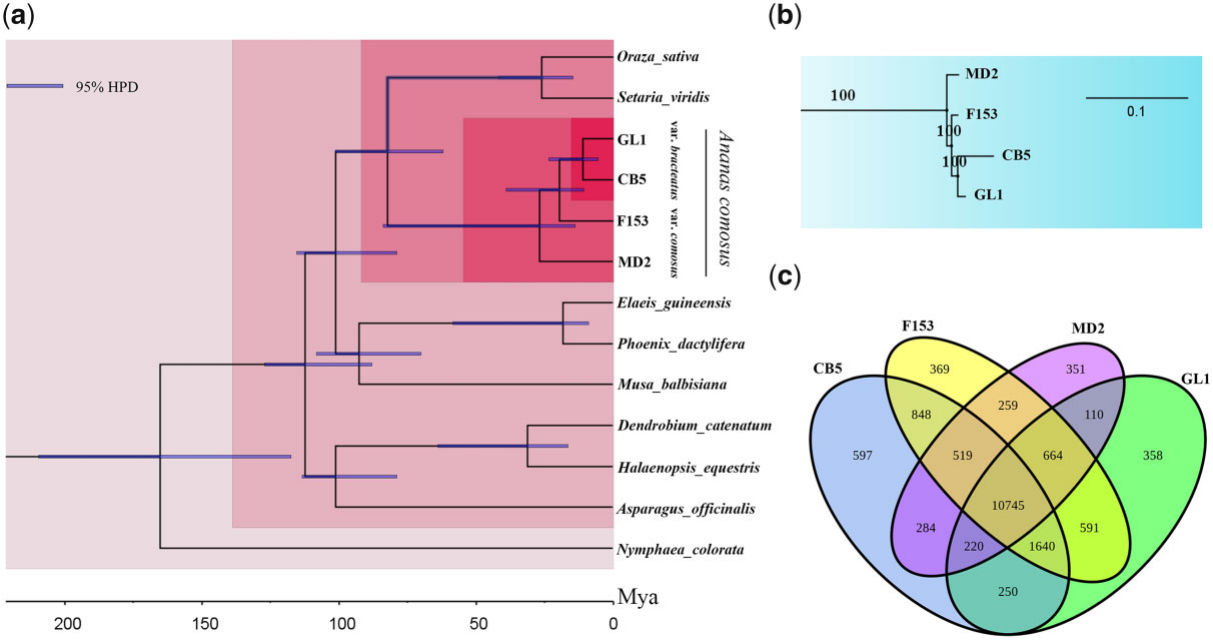
The evolutionary relationships among GL1 and other plant species. a) The phylogenetic tree of 4 pineapples and 9 other representative species. Inferred divergence times (MYA) are denoted at each node. b) Local evolutionary relationships of 4 pineapple varieties. c) The Venn diagram shows the overlap of orthogroups among 4 pineapple assemblies.

We performed a phylogenetic analysis using 4 pineapple assemblies and those of 9 other representative species (Fig. 2a; Supplementary Table 10). A total of 499 shared single-copy orthogroups were used for phylogenetic tree construction and divergence time estimation (Supplementary Table 11 and File 2). Our phylogenetic analysis confirmed the close relationship among GL1, CB5, F153, and MD2. The rates of nucleotide substitutions and their ratios we identified in these accessions show that bromeliads diverged from the nearest common ancestor of the Bromeliaceae and Poaceae ∼82.36 MYA, which was earlier than ∼112 MYA, as indicated by fossil evidence (Kumar et al. 2017). The time tree of the 4 bromeliad varieties we analyzed shows that var. *bracteatus* accession CB5 and var. *comosus* accession F153 diverged ∼19.64 MYA. Accessions CB5 and GL1 diverged ∼10.91 MYA (Fig. 2a). Further, as the branch lengths of the phylogenetic tree represents the cumulative amount of evolution that has taken place in terms of substitutions per nucleotide site, the longer branch lengths leading to the admixed species CB5 indicates a larger cumulative amount of evolution in its lineage compared with those of accessions GL1, F153, and MD2 (Fig. 2b).

Furthermore, we identified 358, 597, 369, and 351 orthogroups unique to the GL1, CB5, F153, and MD2 assemblies, respectively. In addition, 10,745 orthogroups are shared among the 4 pineapple assemblies. A total of 110, 250, and 591 gene families are uniquely shared between GL1 and MD2, between GL1 and CB5, and between GL1 and F153, respectively (Fig. 2c; Supplementary Table 12). The unique gene families among the 4 pineapple assemblies are enriched in a series of GO terms (*P* < 0.05), including “cysteine-type peptidase activity” (GO:0008234), “defense response” (GO:0006952), “chloroplast” (GO:0009507), and “photosynthetic electron transport chain” (GO:0009767) (Supplementary Table 13). In addition, the Ka/Ks ratios for all 499 shared single-copy orthologs were calculated for the GL1, CB5, F153, and MD2 genome assemblies. Totals of 45, 45, 33, and 35 positively selected genes were identified in the GL1, CB5, F153, and MD2 genomes, respectively (Supplementary Table 14). GO term enrichment analysis (*P* < 0.05) of those positively selected genes showed that they are enriched for terms such as “GTP binding” (GO:0005525), “damaged DNA binding” (GO:0003684), and “nucleotidyltransferase activity” (GO:0016779) (Supplementary Table 15).

### Population genetics analysis

**Fig. 3.**
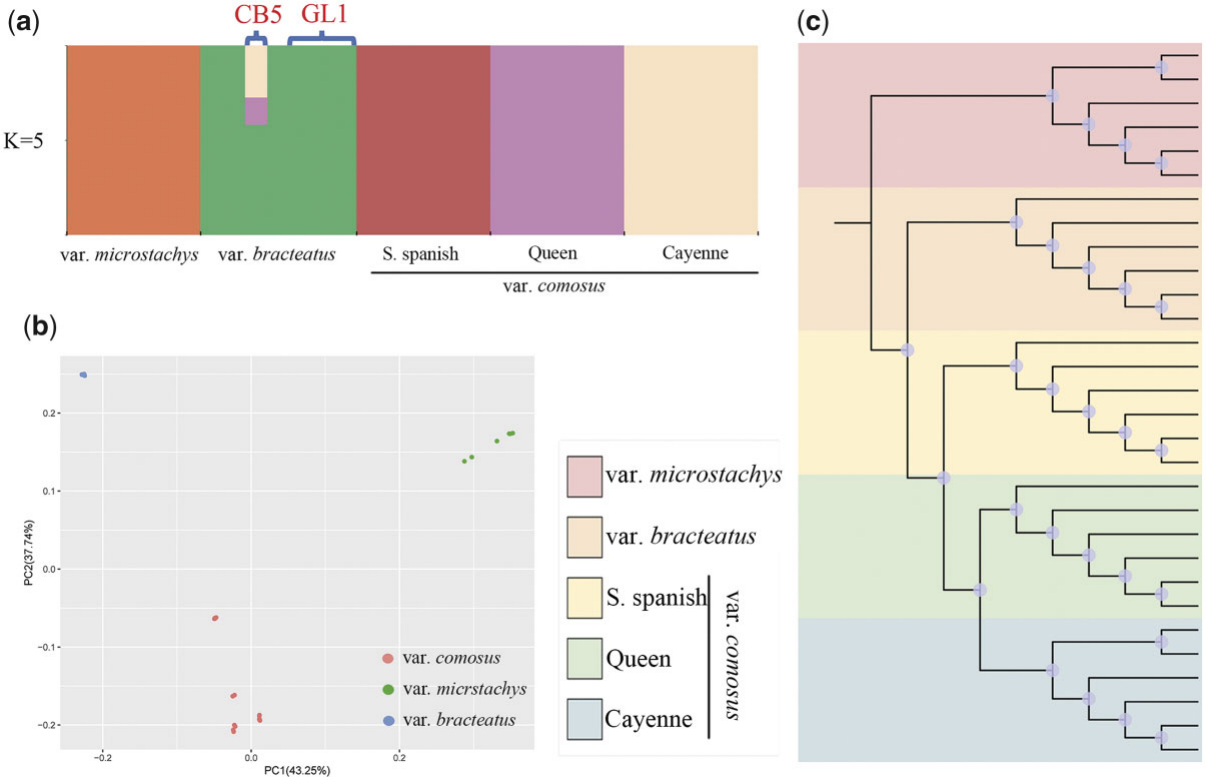
Population genetic analysis of the 31 resequenced pineapple samples. a) Population structure analysis for all 31 pineapple resequencing samples; b) PCA of 30 pineapple samples with no population admixture; c) evolutionary relationships among 30 pineapple samples with no population admixture.

We chose 31 resequenced pineapple samples to perform population genetic analysis. According to the results in the CB5 genome, the 31 pineapple resequencing pineapple samples could be divided into 5 groups, including a group of 6 wild var. *microstachys* accessions, a group of 7 var. *bracteatus* accessions, a group of 6 var. *comosus* cultivar ‘S. spanish (Singapore. spanish)’ samples, a group of 6 var. *comosus* cultivar ‘Queen’ samples, and a group of 6 var. *comosus* cultivar ‘Cayenne’ samples (Chen et al. 2019). After SNP calling, we identified a total of 155,174 high-quality SNPs. We performed a population structure analysis for these samples with *K* values ranging from 2 to 7. (Cross-validation error is shown in Supplementary Fig. 5.) When K = 5, the samples can be clearly divided into 5 groups with the lowest cross-validation error. Our population structure analysis shows that unlike accession CB5, which was derived from admixture of 3 species, we identified no signatures of admixture in the GL1 genome (Fig. 3a). Notably, a previous study showed that accession CB5 resulted from admixture of 4 populations (Chen et al. 2019). This distinction could have been caused by the use of the genome of a different pineapple variety as the reference genome for SNP calling. Pineapple samples with no signature of admixture were then used to perform further analysis. PCA shows that the remaining samples can be clearly divided into 3 groups that represent var. *microstachys* accessions, var. *bracteatus* accessions, and var. *comosus* accessions (Fig. 3b). Our phylogenetic tree shows that the var. *bracteatus* accessions are more closely genetically related to the var. *microstachys* accessions than to the 3 var. *comosus* cultivars. Further, our results indicated that the ‘S. spanish’ cultivar is more closely genetically related to the var. *bracteatus* accessions than are the ‘Queen’ and ‘Cayenne’ cultivars (Fig. 3c). These results agree with those of a previous analysis of the CB5 genome (Chen et al. 2019).

### Genomic variants between the *Ananas* GL1, CB5, and F153 genomes

Genomic variants, including insertions, deletions, inversions, and duplications (Li et al. 2021) are important sources of diversity that can be used for selection and breeding to improve crops. Comparison of the GL1 and CB5 genomes identified 547,215 SNPs and 145,867 INDELs (<100 bp) between them, with an average of 1.85 SNPs and 0.3 INDELs per kilobase (Supplementary Table 17). We identified a total of 4,221 GL1-specific regions covering 3.56 Mb with 158 GL1-specific genes and 10,285 CB5-specific regions covering 9.61 Mb with 393 CB5-specific genes. The longest PAV in the GL1 genome is a 12,700-bp GL1-specific segment from 13,897,201 to 13,909,900 bp on chromosome 24, while the longest CB5-specific segment is a 19,700-bp region on chromosome 25 from 17,155,701 to 17,175,400 bp (Supplementary Table 18).

Comparison of the GL1 and F153 genomes revealed 80,292 SNPs and 12,670 INDELs with an average of 0.17 SNPs and 0.03 INDELs per kb (Supplementary Table 17). Within these data, a total of 11,848 GL1-specific regions covering 9.60 Mb with 227 GL1-specific genes, and 21,841 F153-specific regions covering 26.21 Mb with 735 F153-specific genes were identified (Supplementary Table 19). Further, comparison of the F153 and CB5 genomes reveal 19,615 F153-specific regions covering 28.13 Mb with 203 F153-specific genes, and 16,880 CB5-specific regions covering 13.73 Mb with 280 CB5-specific genes (Supplementary Table 20). In total, 15,973 F153-specific regions are absent from both the GL1 and CB5 genomes, covering 24.18 Mb of the F153 genome and 108 PAV genes. The longest PAV sequence segment that is absent from both the GL1 and CB5 genomes is a 94,200-bp F153-specific segment from 65,001 to 159,200 bp on chromosome 20, but no PAV genes were identified within this region (Supplementary Table 21).

The PAV-specific genes identified between the 3 chromosome-level pineapple genome assemblies were subjected to GO enrichment analysis (*P* < 0.05). The enriched GO terms associated with these PAV genes include “response to red or far red light” (GO:0009639), “defense response” (GO:0006952), and “photosynthesis” (GO:0015979) (Supplementary Table 22).

Notably, compared with the genomes of var. *bracteatus* accessions CB5 and GL1, the genome of var. *comosus* accession F153 contains more accession-specific regions and genes. We also observed that the numbers of SNPs and INDELs between the GL1 and F153 genomes are lower than between the GL1 and CB5 genomes. Further analysis shows that this phenomenon might be due to fewer variants identified by mapping GL1 short reads to the F153 genome. In contrast, the coverage rate for mapping GL1 short reads to CB5 is 95.84% (>4×), a higher rate of coverage rate than for mapping these reads to F153 (87.63%) (Supplementary Table 17). Similarly, when PacBio long reads for GL1 were aligned to the F153 and CB5 genomes, again fewer PAV variants were in F153 than in CB5 (4,310 compared with 105,974). This could have been caused by the larger differences between the GL1 and F153 genomes or due to an incomplete assembly of the F153 genome.

### Comparison of genes related to anthocyanin biosynthesis between pineapple accessions

The red coloration of the fruit, leaves, and bracts in var. *bracteatus* is the main phenotypic difference between this and other pineapple accessions. Anthocyanin biosynthesis is a very important aspect of the colors of the fruit, leaves, and bracts of var. *bracteatus* (Zhou et al. 2021) and for stress response (Sarma et al. 1997; Gould and Quinn 1999; Ahmed et al. 2015). The accumulation of anthocyanin results from phenylpropanoid biosynthesis and flavonoid biosynthesis, and the functions of certain structural genes determine the biosynthesis of anthocyanins. Through our PAV analysis, we found that the genomes of the var. *bracteatus* accessions GL1 and CB5 both contain 2 specific chalcone synthase (CHS) genes, that were absent from the F153 genome. CHS genes play an important role in anthocyanin biosynthesis, by catalyzing the conversion of 4-coumaroyl-CoA to chalcone (Jiang et al. 2008). In GL1, these 2 genes are located on chr14:5,348,639 5,355,578 and chr14:5472048 5,473,967, while in CB5, these 2 genes are located on chr14:7,219,504 7,227,041 and chr14:7,347,253 7,349,144 (Supplementary Table 23).

**Fig. 4.**
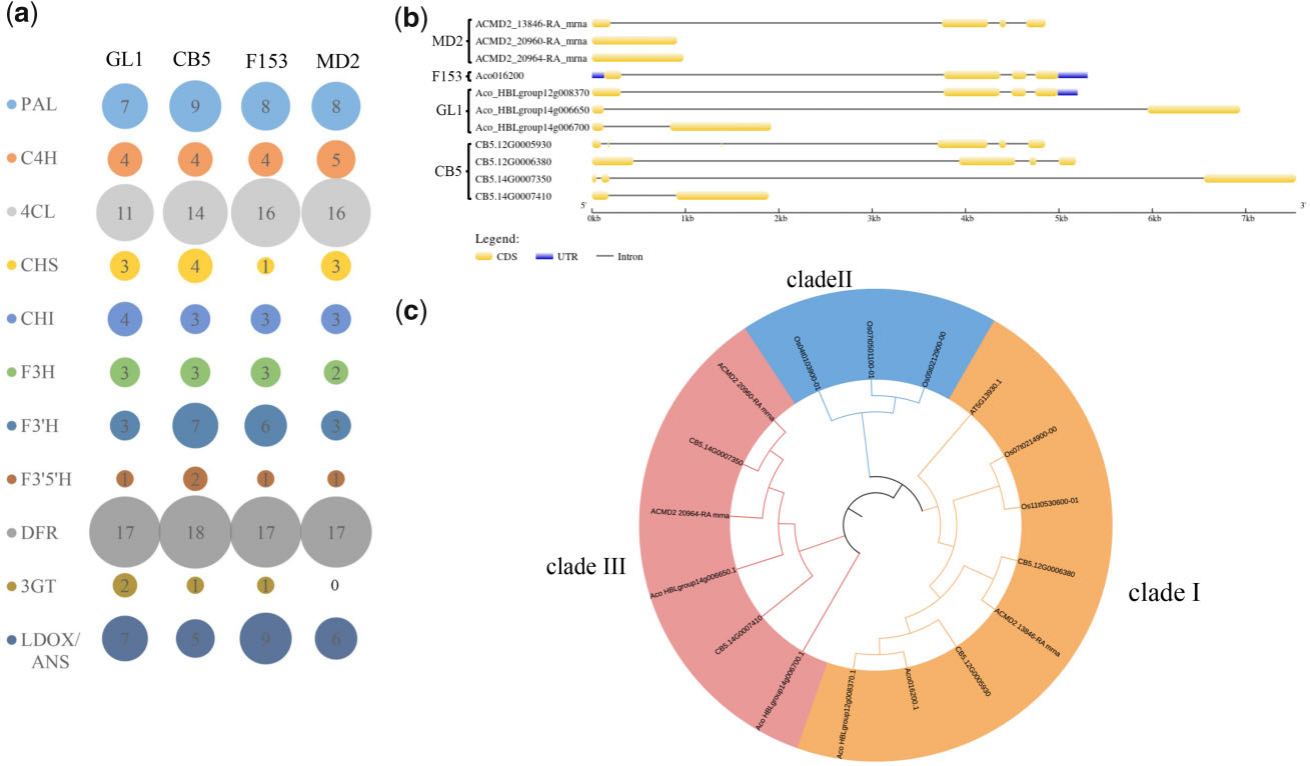
Comparison of genes encoding enzymes related to anthocyanin biosynthesis in 4 pineapple genomes. a) Gene family identification for genes likely involved in anthocyanin biosynthesis; b) the structure of CHS genes in 4 pineapple genomes; c) phylogenetic tree of all CHS genes identified in a comparison of *A. thaliana*, *O. sativa*, and pineapple in the present study.

Genes in the 4 pineapple genomes encoding enzymes related to anthocyanin biosynthesis including phenylalanine ammonia-lyase, cinnamate 4-hydroxylase, 4-coumarate CoA ligase, chalcone synthesis (CHS), chalcone isomerase (CHI), flavanone 3-hydroxylase (F3H), flavonoid 3′-hydroxylase (F3′H), flavonoid 3′5′-hydroxylase (F3′5′H), dihydroflavonol 4-reductase, flavonoid 3-O-glucosyltransferase (3GT), leucoanthocyanidin dioxygenase, and anthocyanidin synthase were identified via local BLAST searches using corresponding genes from *A.* *thaliana* and *O.* *sativa* as queries with the parameters E-value <10^-5^, identity ≥50%, and coverage ≥30% (Supplementary Table 10). Compared with their homologs in the var. *comosus* accessions F153 and MD2, more than half of these genes, including those encoding CHS, CHI, F3H, F3′H, and F3′5′H have undergone expansion in var. *bracteatus* accessions. The most significantly expanded genes are those encoding CHS and flavonoid 3GT, which have expanded 1.74-fold (7 genes compared with 4 genes) and 3-fold (3 genes compared with 1 gene) relative to their homologs in 2 var. *comosus* accessions, respectively (Fig. 4a). Combining the results of our PAV variation analysis and gene family identification, we chose to focus on the CHS gene family for further analysis.

All of the CHS genes identified in the 4 pineapple assemblies were further confirmed by searching the PFAM database (Finn et al. 2014). Except for ACO_HBLGroup14G0067001, which only has the N-terminal domain and lacked the C-terminal domain of CHS, all of the other CDSs that likely encode CHS contain both the C- and N-terminal domains of CHS.

The average amino acid length of CHS-encoding genes in 2 var. *bracteatus* accessions is 387.29 aa, which is larger than that in 2 var. *comosus* accessions (336.50 aa) (Fig. 4b). All CHS-encoding genes in 2 var. *bracteatus* accessions are located on chromosome 12 or 14, while the CHS-encoding genes in var. *comosus* accession F153 are located on chromosome 17. Among the 11 CHS-encoding genes in the 4 pineapple genomes, 8 exist in pairs with 111.58–134.49 kb between them, which suggests that these pairs of CHS-encoding genes might have arisen from tandem duplication (Supplementary Table 23).

We performed phylogenetic analysis of the predicted amino acid sequences of 1 *A.* *thaliana* CHS protein, 5 *O.* *sativa* CHS proteins, and 11 pineapple CHS proteins using MEGA X (Kumar et al. 2018) with the neighbor-joining method and 2,000 bootstraps and found that these CHS genes can be divided into 3 clades. Clade I includes 1 *A.* *thaliana* CHS-encoding gene, 2 *O.* *sativa* CHS-encoding genes, and 5 pineapple CHS-encoding genes from 4 pineapple varieties (GL1, CB5, F153, and MD2). The CHS-encoding genes of GL1 and CB5 cluster in clade I and are both located on chromosome 12, suggesting that these CHS-encoding genes are conserved among these species. Clade II includes only 3 rice CHS-encoding genes, suggesting that these CHS-encoding genes are rice-specific. Clade III is a pineapple-specific clade and includes 6 pineapple CHS-encoding genes from 3 pineapple varieties (GL1, CB5, and MD2) (Fig. 4c). Among these 6 CHS-encoding genes, 2 GL1-specific genes and 2 CB5-specific genes are absent from F153. The other 2 CHS-encoding genes in clade III are from MD2.

In addition, the biosynthesis of anthocyanin shares the phenylpropanoid biosynthesis pathway with lignin biosynthesis, so the biosynthesis of lignin can affect the biosynthesis of anthocyanin. Our PAV analysis showed that a gene encoding a putative hydroquinone glucosyltransferase (AS) (Aco_HBLgroup8g007190) is specific to GL1 and absent from the CB5 genome. According to its UniProtKB annotation, AS is a broad spectrum multifunctional glucosyltransferase that functions in lignin biosynthesis (Boutet et al. 2007). Meanwhile, a gene encoding the transcription factor MYB2 (Aco_HBLgroup14g000300) was also identified as specific to the GL1 genome and could play a regulatory role in lignin biosynthesis (Wang et al. 2004; Goicoechea et al. 2005) and tolerance to salt, cold, and drought stresses (Yang et al. 2012). The GL1-specific genes identified here might play important roles in anthocyanin biosynthesis and stress responses in pineapple (Supplementary Table 23).

### PAV genes related to chimeric character of the leaves

Chlorophyll and chloroplasts are essential for photosynthesis. Our previous study found that the albino leaf margins of GL1 result from the absence of chlorophyll and incomplete development of chloroplasts (Xue et al. 2019; Mao et al. 2020). By comparing the GL1 and CB5 genomes, 158 GL1-specific genes were found in the GL1 genome assembly and 393 GL1-absent genes were found in the CB5 genome assembly. The GL1-specific genes include those encoding the transcription factor Golden2-like protein 1 (GLK1) (Aco_HBLgroup14g003910), Squamosa promoter-binding-like protein 8 (SPL8) (Aco_HBLgroup16g007730), Protein terminal ear1 homolog (PLA2) (Aco_HBLgroup17g012350), and FT-interacting protein 4 (FTIP4) (Aco_HBLgroup20g001900), which are related to chlorophyll biosynthesis, chloroplast development, photosynthesis, and leaf development, respectively (Supplementary Table 23). Transcription factor GLK1 acts as an activator of nuclear photosynthetic genes involved in chlorophyll biosynthesis, light harvesting, and electron transport (Fitter et al. 2002; Waters et al. 2009). The transcription factor SPL8 controls ligule and auricle development during the development of the laminar joint at the boundary between the leaf blade and sheath (Lee et al. 2007). PLA2 might regulate leaf initiation rates and vegetative phase duration (Kawakatsu et al. 2006). Proliferating and differentiating shoot stem cells in plant shoot apical meristems (SAMs) require FTIP4 to control the dynamics of their maintenance or differentiation into other plant organs by controlling STM localization in and trafficking between SAM cells (Liu et al. 2018).

**Fig. 5.**
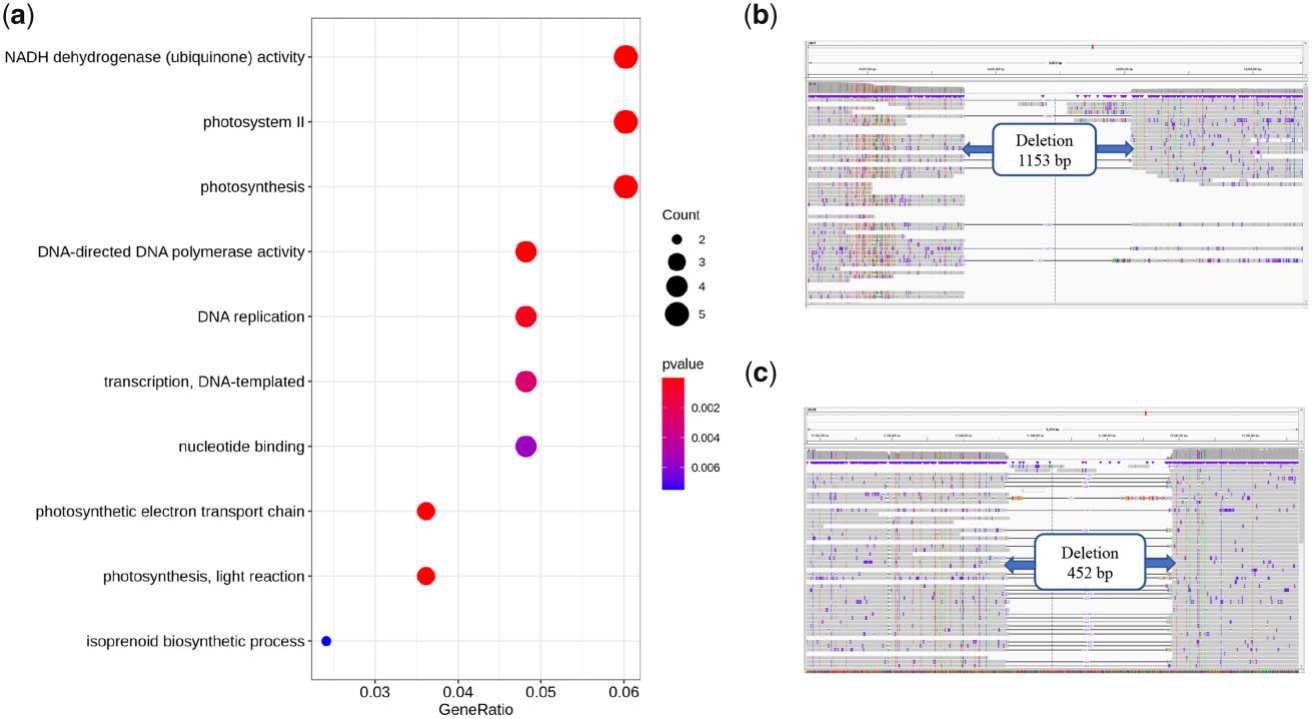
Analysis of GL1-absent genes in *Ananas* accession CB5. a) Top 10 significantly enriched (*P* < 0.05) GO terms associated with GL1-absent genes in CB5. b) Absent segment that would contain a photosystem antenna protein-like protein gene. c) Absent segments that would contain 2 psbK genes.

Further, our GO term enrichment analysis of GL1-absent genes showed enrichment of certain GO terms related to photosynthesis, such as “photosystem II (PSII)” (GO:0009523), “photosynthesis” (GO:0015979), and “photosynthetic electron transport chain” (GO:0009767) (Fig. 5a; Supplementary Table 22). Terms for 5 PSII reaction center-related proteins (CB5.25G0008260, CB5.25G0008500, CB5.25G0008830, CB5.25G0009210, and CB5.25G0009250) were also found among these enriched GO terms. The PAV sequences identified by aligning the GL1 PacBio long reads to the CB5 genome were subjected to local BLAST searches of the PAV genes identified by comparison of the GL1 and CB5 genomes (e-value <1e−5, identity >80%, coverage >20%). The results showed that 2 specific segments in CB5 that are absent in GL1 include 3 photosynthesis-related genes. One 1153-bp segment is located on chromosome 7 beginning at 14,691,758 bp in the CB5 genome. This segment contains a gene putatively encoding photosystem antenna protein-like protein (CB5.25G0009250), which plays roles in chlorophyll binding and photosynthetic electron transport in PSII (Fig. 5b; Supplementary Table 23). Another segment absent from the CB5 genome is located on chromosome 20 at 12,548,728 bp, with a total length of 452 bp. This segment contains 2 genes encoding PSII reaction center protein K (psbK) proteins (CB5.25G0009210 and CB5.25G0008830) (Fig. 5c; Supplementary Table 23). According to the annotation at the UniProtKB database, psbK is 1 component of the core complex of PSII and is required for the assembly and/or stability of PSII (Boutet et al. 2007). PSII is a complex of pigments and proteins within the thylakoid membrane that catalyzes the primary photochemical reaction and the release of oxygen, protons, and electrons through the decomposition of water during photosynthesis (Bibby et al. 2003). Thus, these GL1-specific and GL1-absent genes might play roles in or result from the formation of GL1 chimeric leaf features.

## Data availability

The raw genome sequencing data, the final chromosome assembly, and genome annotation have been deposited into CNGB sequence archive (CNSA) of China National GeneBank DataBase (CNGBdb) under BioProject number CNP0001166, and the biosample for GL1 assembly is CNS0254294. The raw genome sequencing data and the final chromosome assembly have also been submitted to National Center for Biotechnology Information (NCBI) under BioProject number PRJNA747096, and the biosample for GL1 assembly is SAMN20254283. The supplementary material includes supplementary tables, figures, and files. The isoforms used as full-length transcripts are available in Supplementary File 1. Orthogroup gene families from OrthoFinder analysis are available in Supplementary File 2. Vcf file of SNPs information for population genetic analysis are available in Supplementary File 3. Supplementary files are available at figshare: https://doi.org/10.25387/g3.16961116. The plant materials are cultivated in Sichuan Agriculture University.
